# Successful Pregnancy in a Woman With Primary Infertility Associated With Isolated Hypogonadotropic Hypogonadism and Partial Empty Sella Syndrome: A Case Report

**DOI:** 10.7759/cureus.109372

**Published:** 2026-05-21

**Authors:** Dina Houjjaj, Sara Mouhmouh, Amal Benbella

**Affiliations:** 1 Gynecology and Obstetrics, Les Orangers University Hospital for Maternity and Reproductive Health, Rabat, MAR; 2 Gynecology and Obstetrics, Mohammed VI University Hospital, Agadir, MAR

**Keywords:** empty sella syndrome, gonadotropin therapy, hypogonadotropic hypogonadism, ovarian response, primary infertility

## Abstract

Empty sella syndrome (ESS) is a radiological condition characterized by herniation of cerebrospinal fluid into the sella turcica, resulting in compression of the pituitary gland. Although often incidental, ESS may be associated with selective pituitary hormone deficiencies, including gonadotropin deficiency, leading to hypogonadotropic hypogonadism (HH) and infertility. We report the case of a 34-year-old woman with 14 years of primary infertility and a history of primary amenorrhea. Hormonal assessment revealed profound gonadotropin deficiency with a follicle-stimulating hormone (FSH) of 0.39 IU/L, luteinizing hormone (LH) less than 0.10 IU/L, and estradiol below 5 pg/mL, with preserved thyroid and prolactin function. Anti-Müllerian hormone (AMH) level was 0.8 ng/mL. Pituitary magnetic resonance imaging (MRI) demonstrated a partial empty sella without adenoma. Hysterosalpingography and semen analysis were normal. Ovulation induction with human menopausal gonadotropin resulted in pregnancy after one stimulation cycle. Cesarean section was performed at 38+3 weeks of gestation, delivering a healthy male newborn weighing 3180 g with an Apgar score of 10/10. Partial empty sella syndrome may be associated with isolated central hypogonadism leading to infertility, and individualized gonadotropin therapy can restore ovulation and achieve a successful pregnancy outcome.

## Introduction

Empty sella syndrome (ESS) is defined by partial or complete filling of the sella turcica with cerebrospinal fluid, leading to compression and flattening of the pituitary gland [1]. ESS may be primary, related to a congenital defect of the diaphragma sellae, or secondary to pituitary surgery, radiation, infarction, or trauma [1]. Partial empty sella is a common neuroimaging finding, identified in up to 12-35% of cranial magnetic resonance imaging (MRI) examinations performed for various indications, and is most often clinically silent [2]. In the majority of cases, the anterior pituitary preserves normal endocrine function despite the radiological appearance, and the finding is therefore frequently regarded as incidental [3].

Only a minority of patients develop biologically demonstrable hypopituitarism, and among these, selective involvement of the gonadotropic axis with full preservation of the other anterior pituitary axes is a particularly uncommon endocrine phenotype [3,4]. The pathophysiological link between partial empty sella and isolated hypogonadotropic hypogonadism (IHH) is thought to arise from a weakness of the diaphragma sellae, which normally shields the pituitary gland from the overlying subarachnoid space. When this barrier is incompetent, cerebrospinal fluid herniates into the sella turcica and progressively flattens the anterior pituitary against the sellar floor. Gonadotroph cells appear particularly vulnerable to this chronic mechanical compression, leading to preferential impairment of pulsatile luteinizing hormone (LH) and follicle-stimulating hormone (FSH) secretion, while thyrotroph, lactotroph, and corticotroph functions are often preserved [2,4]. The resulting gonadotropin deficiency disrupts ovarian folliculogenesis, producing hypoestrogenism, primary or secondary amenorrhea, anovulation, and infertility [5]. Nevertheless, the relationship between partial empty sella and isolated hypogonadotropic hypogonadism should be interpreted as a clinically relevant association rather than as a firmly established causal link, since a direct mechanistic relationship is difficult to demonstrate in individual cases.

Ovulation induction using exogenous gonadotropins remains the cornerstone of fertility management in women with central hypogonadism [6]. However, published data specifically addressing fertility outcomes in women with partial empty sella and isolated central hypogonadism remain limited and are largely restricted to isolated case reports and small series [1,5]. The optimal stimulation strategy, the predictive value of ovarian reserve markers, and the obstetric course in this subgroup remain poorly characterized. Reporting well-documented cases is essential to progressively build the body of evidence required to guide clinical decision-making in this rare context.

We report a case of long-standing primary infertility in a woman with isolated gonadotropic deficiency occurring in association with partial empty sella syndrome, in whom individualized gonadotropin stimulation resulted in pregnancy after a single cycle and uneventful term delivery by cesarean section.

## Case presentation

A 34-year-old woman presented to our department for evaluation of primary infertility of 14 years' duration. She reported a history of primary amenorrhea, with no past medical or surgical history and no history of head trauma, pituitary surgery, or radiation exposure. Clinical examination revealed a body mass index of 28 kg/m². Secondary sexual characteristics were fully developed, corresponding to Tanner stage S5P5 [7]. The Tanner staging system is in the public domain and freely available for clinical use without a license. There was no galactorrhea, visual disturbance, or neurological abnormality.

The hormonal profile, summarized in Table 1, illustrates the characteristic dissociation between a profoundly impaired gonadotropic axis and the full preservation of the other anterior pituitary axes, which constitutes the biological signature of the reported phenotype. The findings were consistent with isolated gonadotropic deficiency, with profoundly suppressed LH and FSH concentrations alongside undetectable estradiol, while prolactin and thyroid-stimulating hormone (TSH) remained within normal limits. Anti-Müllerian hormone (AMH) level was 0.8 ng/mL, suggesting reduced but present ovarian reserve.

**Table 1:** Baseline hormonal laboratory findings grouped by pituitary axis. FSH: follicle-stimulating hormone; LH: luteinizing hormone; TSH: thyroid-stimulating hormone; AMH: anti-Müllerian hormone. Reference ranges are laboratory-specific and correspond to the follicular phase of the menstrual cycle where applicable. Hormonal assays were performed using the standard chemiluminescent immunoassay methodology of our institutional reference laboratory. Abnormal values are indicated with downward arrows.

Pituitary axis	Parameter	Patient value	Reference range	Interpretation
Gonadotropic axis	FSH	0.39 IU/L	3.5–12.5 IU/L (follicular phase)	Low ↓
	LH	<0.10 IU/L	2.4–12.6 IU/L (follicular phase)	Low ↓
	Estradiol (E2)	<5 pg/mL	12.4–233 pg/mL (follicular phase)	Low ↓
	Progesterone	0.28 ng/mL	0.15–1.4 ng/mL (follicular phase)	Normal
Lactotropic axis	Prolactin	6.82 ng/mL	2.0–29.0 ng/mL	Normal
Thyrotropic axis	TSH	1.02 mIU/L	0.4–4.0 mIU/L	Normal
Corticotropic axis	Morning cortisol	Within normal range	Institutional laboratory range	Normal
Ovarian reserve	AMH	0.8 ng/mL	1.0–3.5 ng/mL (age 30–35 years)	Reduced ↓

A systematic evaluation was undertaken to exclude alternative causes of hypogonadotropic hypogonadism (HH) before attributing the gonadotropic deficiency to the partial empty sella. Functional hypothalamic causes were considered unlikely given the patient's stable body mass index of 28 kg/m², absence of excessive physical activity, no history of eating disorder or significant psychological stress, and no recent weight fluctuation. Hyperprolactinemia was ruled out by a normal serum prolactin concentration (6.82 ng/mL), and primary or central thyroid dysfunction was excluded by a normal TSH (1.02 mIU/L) together with normal free thyroxine values. Adrenal insufficiency was clinically excluded by the absence of suggestive symptoms (asthenia, hypotension, hyperpigmentation, electrolyte disturbances), and morning cortisol measurement was within the normal range, making a coexisting corticotropic deficiency unlikely. Hemochromatosis and other infiltrative disorders were considered improbable given the absence of relevant personal or family history, normal serum ferritin and transferrin saturation, and an unremarkable liver profile. There was no clinical or biological evidence of chronic systemic illness, autoimmune disease, or malnutrition. The patient had no anosmia or hyposmia, no midline craniofacial anomaly, no synkinesia, and no relevant family history of delayed puberty or infertility, making Kallmann syndrome and other forms of congenital hypogonadotropic hypogonadism clinically unlikely; formal genetic testing was not available in our setting, which constitutes an acknowledged limitation. Finally, the patient had no history of head trauma, cranial irradiation, pituitary surgery, neurosurgical intervention, or peripartum hemorrhage, allowing acquired secondary causes of pituitary insufficiency to be excluded.

Plasma adrenocorticotropic hormone (ACTH) and dynamic stimulation tests of the corticotropic axis were not performed, as they were not considered clinically indicated in this asymptomatic patient with a reassuring basal cortisol value. Likewise, insulin-like growth factor 1 (IGF-1) measurement and dynamic evaluation of the somatotropic axis were not performed, given the absence of clinical evidence of adult growth hormone deficiency and no immediate therapeutic implications in the context of fertility management. The diagnosis of isolated hypogonadotropic hypogonadism occurring in association with partial empty sella syndrome was, therefore, retained on the basis of a profound and unequivocal gonadotropic deficiency contrasting with clinically and biologically preserved thyrotropic, lactotropic, and corticotropic axes, while acknowledging that a more exhaustive endocrine profiling, including ACTH, IGF-1, and dynamic pituitary testing, would have provided additional formal confirmation.

**Figure 1:**
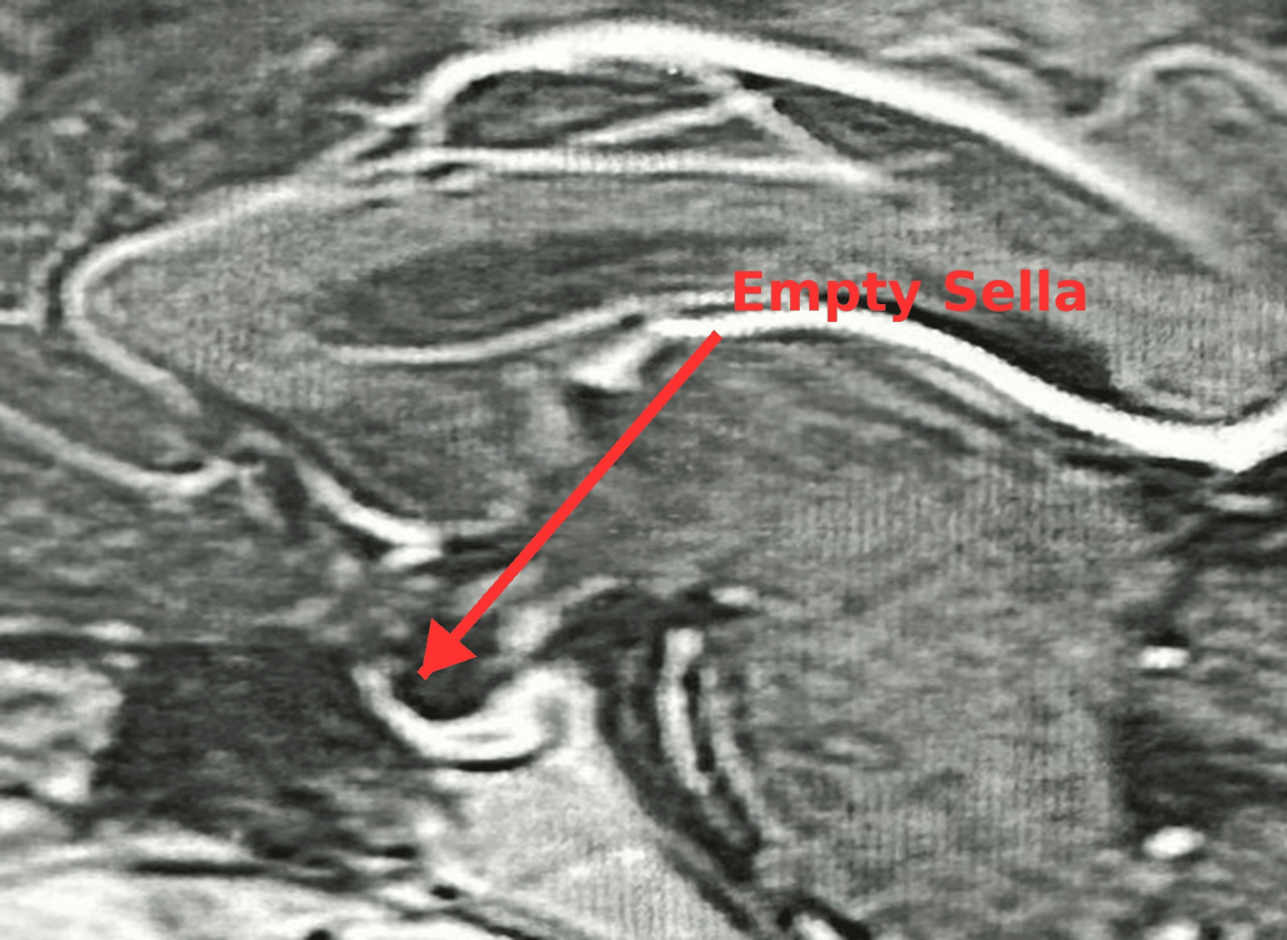
Pituitary MRI (sagittal T1-weighted view) demonstrating partial empty sella syndrome. Pituitary MRI, sagittal T1-weighted view, demonstrating partial empty sella syndrome. The red arrow indicates the region of cerebrospinal fluid herniation into the sella turcica, with flattening and thinning of the anterior pituitary gland against the sellar floor. The posterior pituitary bright spot and the pituitary infundibulum are preserved and in a normal midline position. No focal intrasellar or parasellar lesion, microadenoma, or suprasellar extension was identified. These features are characteristic of a partial primary empty sella, with no associated structural pituitary pathology.

Pituitary MRI demonstrated herniation of the subarachnoid space into the sella turcica with thinning of the anterior pituitary gland and preserved posterior pituitary signal, without evidence of microadenoma, as illustrated in Figure 1. The findings were consistent with partial primary empty sella syndrome.

Hysterosalpingography revealed a normal uterine cavity and bilateral tubal patency. The husband's semen analysis was within normal limits according to the WHO 2021 reference criteria [8]. A diagnosis of primary infertility of central origin in association with isolated hypogonadotropic hypogonadism and partial ESS was therefore established.

Taken together, this case illustrates a distinctive and reproducible clinical pattern that may help clinicians identify similar presentations: a young woman with long-standing primary amenorrhea and primary infertility, fully developed secondary sexual characteristics, profoundly suppressed LH, FSH, and estradiol concentrations contrasting with preserved prolactin, thyroid, and corticotropic function, a reduced but not abolished AMH level, normal hysterosalpingography and normal partner semen analysis, and a partial empty sella on pituitary MRI without evidence of adenoma or other structural lesion. Recognition of this constellation should prompt a structured pituitary workup and consideration of gonadotropin-based ovulation induction, since the reproductive prognosis--as illustrated here--can be excellent despite an apparently unfavorable baseline biological profile.

Management and outcome

Ovulation induction was initiated using human menopausal gonadotropin (hMG), providing both FSH and LH activity. Adequate follicular development was achieved, and pregnancy occurred after a single stimulation cycle. The pregnancy was regularly monitored and progressed without endocrine or neurological complications. At 38 weeks and three days of gestation, cesarean section was performed, and a healthy male newborn weighing 3180 g was delivered with an Apgar score of 10/10 at one and five minutes [9]. The postoperative and postpartum courses were uneventful.

## Discussion

Empty sella syndrome is primarily a radiological entity defined by herniation of the subarachnoid space into the sella turcica, resulting in compression and flattening of the pituitary gland [1]. Its incidence on neuroimaging is estimated at approximately 12%, rising to 35% in clinical practice, and endocrine pituitary disorders are reported in 19% to 40% of patients [2]. A pooled meta-analysis found hypopituitarism in 52% of cases, with multiple pituitary hormone deficiencies in 30% and isolated deficiencies in 21%; growth hormone and gonadotropins were the most common isolated insufficiencies [3]. Selective involvement of the gonadotropic axis with sparing of other pituitary axes has been attributed to the particular anatomical vulnerability of gonadotroph cells to mechanical compression within the anterior pituitary [2,4].

In the present case, profoundly suppressed LH and FSH concentrations alongside intact prolactin, TSH, and clinically and biologically preserved corticotropic function were consistent with isolated central hypogonadism rather than panhypopituitarism. This dissociation is well recognized in partial ESS and supports the concept of selective compressive injury to the gonadotroph cell population [4]. Notably, the most common condition associated with primary empty sella is idiopathic intracranial hypertension (IIH), which classically affects women of childbearing age with obesity, and over 70% of patients with IIH are found to have empty sella on MRI [2]. Although our patient had no overt features of IIH, the contribution of subclinical intracranial pressure elevation to gonadotroph dysfunction cannot be excluded and warrants consideration in similar cases [2].

HH results in impaired folliculogenesis, anovulation, and infertility due to insufficient gonadotropin stimulation of the ovaries [5]. Ovulation induction may be achieved through pulsatile gonadotropin-releasing hormone (GnRH) administration or exogenous gonadotropin therapy [6]. The two-cell, two-gonadotropin model of folliculogenesis underscores the obligatory requirement for both FSH--to stimulate granulosa cell aromatase activity and follicular maturation and LH--to drive theca cell androgen synthesis and trigger ovulation [6]. As women with HH lack endogenous LH, combined gonadotropin preparations, such as hMG, are required to replace the absent endogenous hormones and achieve adequate follicular development [10].

A notable finding in our patient was the reduced serum AMH of 0.8 ng/mL, which might initially suggest diminished ovarian reserve. However, the satisfactory follicular response to stimulation highlights a critical limitation of AMH as a prognostic marker in the context of central hypogonadism. There is evidence that AMH production is gonadotropin-dependent and that chronic gonadotropin deficiency suppresses the growing follicular pool, such that both AMH and antral follicle count may significantly underestimate true ovarian reserve in patients with isolated HH [10]. AMH concentration has been shown to increase during hMG stimulation as gonadotropin-dependent follicles are recruited, demonstrating that baseline AMH in IHH reflects only the growing follicular pool rather than the underlying primordial pool [11]. Clinicians should therefore exercise caution when using AMH to counsel patients with central hypogonadism regarding reproductive prognosis, as this marker may substantially underestimate their true fertility potential.

The successful achievement of pregnancy after a single stimulation cycle, despite a borderline AMH level and 14 years of gonadotropin deficiency, reinforces this principle. Studies comparing women with HH to controls undergoing in vitro fertilization (IVF) have demonstrated that, despite requiring longer stimulation and higher total gonadotropin doses, ovarian response and reproductive outcomes, including fertilization rate and live birth rate per cycle, are not significantly different [11]. This supports an optimistic approach to fertility management in women with central hypogonadism, provided adequate gonadotropin supplementation is ensured.

Regarding the pregnancy course, physiological pituitary enlargement during pregnancy and lactation is a recognized phenomenon followed by spontaneous regression; this transient increase in pituitary volume is generally well tolerated in the context of partial ESS, where sellar architecture is only partially compromised [3]. In our patient, pregnancy progressed uneventfully without neurological, visual, or new endocrine complications, consistent with published experience in women with partial ESS and no pre-existing intracranial hypertension [4]. Periodic clinical monitoring for signs of evolving hypopituitarism or increased intracranial pressure during gestation is nonetheless advisable in this population [2].

Limitations

A limitation of the present report is the absence of plasma ACTH measurement, IGF-1 determination, and dynamic pituitary testing, which would have allowed a more exhaustive and formally complete characterization of all anterior pituitary axes. The diagnosis of isolated gonadotropic deficiency, therefore, rests on the combination of a profound biological gonadotropic deficit, normal basal prolactin, TSH, and morning cortisol concentrations, and the absence of clinical features of corticotropic or somatotropic insufficiency, which we acknowledge represents a clinically reasonable but not exhaustive endocrine workup. Genetic testing for congenital forms of hypogonadotropic hypogonadism, including Kallmann syndrome, was not available in our setting and constitutes an additional limitation. Finally, as with any single-case observation, the conclusions drawn from this report cannot be generalized without confirmation in larger cohorts.

## Conclusions

Partial empty sella syndrome represents an underrecognized but potentially treatable cause of isolated hypogonadotropic hypogonadism and long-standing primary infertility, even in the absence of global hypopituitarism or neurological manifestations. The selective impairment of the gonadotropic axis with preservation of other pituitary axes constitutes a distinct endocrine phenotype that should prompt pituitary MRI evaluation in any woman presenting with primary amenorrhea and unexplained infertility of central origin. As illustrated by the present case, serum AMH should be interpreted with particular caution in women with central hypogonadism, since chronic gonadotropin deficiency may suppress the growing follicular pool and lead to an underestimation of the true ovarian reserve. Although this observation cannot, by itself, establish a general rule from a single case, it suggests that a reduced AMH value should not, in this specific clinical context, be considered sufficient grounds to prematurely dismiss the reproductive potential of such patients, and that an individualized stimulation strategy may yield a favorable outcome in selected cases.

A satisfactory ovarian response and term pregnancy can be achieved with individualized combined FSH/LH gonadotropin therapy, even following prolonged amenorrhea and despite a borderline ovarian reserve marker. Partial empty sella syndrome should no longer be regarded as a mere incidental radiological finding; when associated with gonadotropic deficiency, it warrants a structured endocrine workup, a patient-centered stimulation strategy, and close multidisciplinary coordination between endocrinologists, reproductive medicine specialists, and obstetricians. Larger observational series and prospective studies specifically focused on women with partial empty sella syndrome and isolated gonadotropic deficiency are needed to formally confirm whether the favorable reproductive outcome observed in our patient can be replicated at a population level, and to define the optimal stimulation protocol and the most reliable predictive markers of ovarian response in this rare subgroup.
